# Clinical profile of tuberous sclerosis complex patients with and without epilepsy: a need for awareness for early diagnosis

**DOI:** 10.1055/s-0042-1758456

**Published:** 2022-12-19

**Authors:** Conceição Campanario da Silva Pereira, Felipe Diego Gomes Dantas, Maria Luiza Giraldes de Manreza

**Affiliations:** 1 Universidade de São Paulo, Hospital das Clinicas de São Paulo, Departamento de Neurologia Infantil, São Paulo SP, Brazil.; 2 Universidade de São Paulo, Hospital das Clínicas de São Paulo, Departamento de Neurorradiologia, São Paulo SP, Brazil.

**Keywords:** Tuberous Sclerosis, Epilepsy, Early Diagnosis, Neurologic Manifestations, Neuropsychiatry, Esclerose Tuberosa, Epilepsia, Diagnóstico Precoce, Manifestações Neurológicas, Neuropsychiatry

## Abstract

**Background** Tuberous sclerosis complex (TSC) is a multisystemic disorder. Its clinical features manifest differently in several organs, prompting the need for better knowledge.

**Objective** The goal of the present study is to evaluate the neurological findings of TSC, such as cerebral lesions and epilepsy, and to raise awareness of non-neurological findings that could contribute to an earlier diagnosis and treatment.

**Methods** This was a natural history study of patients with a definitive diagnosis of TSC who were referred to a specialized outpatient clinic and followed-up for 2 years with clinical and radiological exams.

**Results** A total of 130 TSC patients (59 males [45.4%], mean age 20.4 years old [1 to 56 years old]); 107 patients (82.3%) were diagnosed with epilepsy. Seizures predominantly began at < 1 year old (72.8%); focal seizures predominated (86.9%); epileptic spasms occurred in 34.5% of patients, and refractory epilepsy was present in 55.1%. Neuropsychiatric disorders, cortical tubers and cerebellar tubers were significantly more frequent in the epilepsy group. Moreover, rhabdomyomas were significantly more frequent in the epilepsy group ( *p* = 0.044), while lymphangioleiomyomatosis was significantly less frequent in the epilepsy group ( *p* = 0.009). Other non-neurological findings did not differ significantly between the groups with and without epilepsy.

**Conclusions** The present study of TSC patients demonstrated the predominantly neurological involvement and significantly higher proportion of TSC-associated neuropsychiatric disorders in the epilepsy group. Higher proportions of cortical and cerebellar tubers may be a risk factor for epilepsy and neurodevelopmental disorders.

## Resumo

**Antecedentes** O Complexo da esclerose tuberosa (CET) é uma doença multissistêmica. As apresentações clínicas em diferentes órgãos são diversas, necessitando um maior conhecimento da doença.

**Objetivo** O objetivo do presente estudo foi avaliar na CET o envolvimento neurológico, como lesões cerebrais e epilepsia, e chamar a atenção para achados não neurológicos que contribuiriam para o diagnóstico e tratamento precoces.

**Métodos** Estudo de história natural do CET em pacientes com critérios diagnósticos definidos encaminhados aleatoriamente para serviço especializado e que foram acompanhados, durante 2 anos, com exames clínicos e radiológicos.

**Resultados** O total de 130 pacientes (59 do sexo masculino [45.4%]), idade média de 20,4 anos [1 a 56 anos]) foram avaliados; 107 pacientes (82.3%) foram diagnosticados com epilepsia. As crises epilépticas se iniciaram especialmente em pacientes < 1 ano de idade (72,8%); predomínio de crise focal (86,9%); ocorrência de espasmos infantis em 34,5% deles e de epilepsia refratária em 55,1%. A frequência de distúrbios neuropsiquiátricos, túberes corticais e túberes cerebelares foi significativamente mais frequente no grupo com epilepsia. Além disso, rabdomioma foi significativamente mais frequente no grupo com epilepsia ( *p* = 0,044), enquanto a linfoangioleiomiomatose foi significativamente menos frequente ( *p* = 0,009). Outros comprometimentos não neurológicos, como os oftalmológicos e os nefrológicos, não diferiram significativamente nos grupos com e sem epilepsia.

**Conclusões** O presente estudo com pacientes com CET com e sem epilepsia pode demonstrar uma maior proporção significativa de transtornos neuropsiquiátricos associados ao CET no grupo com epilepsia. A maior proporção de túberes corticais e cerebelares parecem ser um fator de risco para epilepsia e para o comprometimento do neurodesenvolvimento.

## INTRODUCTION

Tuberous sclerosis complex (TSC) is a rare genetic disease characterized by the involvement of several systems that affects ∼ 1 million people worldwide. 1 It has a prevalence of approximately 1 in 6,000 newborns 2 and an incidence of 1/6,000-10,000 live births annually. 3

It is an autosomal dominant disorder which its neuropathological findings were first described by Bourneville in 1880, when he observed masses in both kidneys in a child with epilepsy. 4

With the advance of molecular techniques, *TSC1* and *TSC2* were cloned in the 1990s, and genotype-phenotype correlations were made possible. 1 Tuberous sclerosis complex is caused by mutations in *TSC1* 5 at 9q34, which produces hamartin, 6 and mutations in TSC2 at 16p13, 7 which produces tuberin. 8 Both proteins act as tumor suppressors. 9

The *TSC1* mutations are more common in familial cases, while mutations in *TSC2* are more likely to be germline mutations and are associated with greater disease severity. Despite the high penetrance of these mutations, their expressivity is variable, which means that every patient with the mutation will have the disease, although its presentation will vary. In ∼ 60 to 70% of affected patients, TSC occurs *de novo* as the result of a spontaneous germline mutation. 10

Among the most severe cases, the proportion of patients with a *de novo TSC2* pathogenic variant is higher than the proportion with *TSC1* mutations. In contrast, in familial cases, the proportions of pathogenic variants of *TSC2* and *TSC1* are almost equal. 11

Patients harboring pathogenic *TSC* variants should undergo individual screening for potentially affected organs, as there is no such thing as an asymptomatic carrier of TSC. 12

The clinical characteristics of the disease include skin, brain, kidney, heart, and lung abnormalities. Central nervous system (CNS) lesions are the leading source of morbidity and mortality, followed by renal diseases. 13 Among neurological symptoms, epilepsy has the greatest impact on quality of life. Early detection of patients at higher risk of seizures as well as their specific seizure types may lead to a better therapeutic response. Moreover, it may be possible to provide preventive epilepsy intervention to TSC patients. 14 Knowledge of the pathophysiology of TSC has informed targeted intervention strategies. 14 15

Tuberous sclerosis complex is associated with neuropsychiatric manifestations at various levels, also known as tuberous sclerosis-associated neuropsychiatric disorders (TAND), which may encompass autism, intellectual disability, attention deficit with or without hyperactivity, depressive disorders, and anxiety. 16

These neuropsychiatric manifestations may have little, moderate, or severe impact on the lives of patients. Additionally, TAND can vary along the life span of the patient and may be minimal or absent in younger patients. 17

Clinicians should be aware of the clinical spectrum of TSC, 18 19 particularly given that an early diagnosis may ensure better outcomes. Thus, studies increasing TSC awareness, 20 such as studies addressing the wide range of TSC-related manifestations and natural history studies, are notably valuable.

The goal of the present study is to raise awareness of neurological and non-neurological TSC manifestations that could help with earlier diagnosis and appropriate treatment. Comparison between groups of TSC patients with and without epilepsy are also provided, improving our understanding of each specific group.

## METHODS

This was a natural history study conducted at the ambulatory clinic for TSC at the Hospital das Clínicas de São Paulo, São Paulo, state of São Paulo, Brazil, from January 2019 to December 2020.

Patients with a definite diagnosis of TSC underwent clinical and radiological exams and were assessed by different specialties, including neurology, nephrology, ophthalmology, dermatology, and psychiatry.

Tuberous sclerosis complex was diagnosed according to established diagnostic criteria. 21 Information about TAND was collected using a standardized checklist. 16

These patients were seen periodically twice yearly and followed-up for 2 years as part of the study. Ancillary tests were performed according to our internal surveillance protocol, and according to the individual needs of each patient. For the present study, we included retrospective relevant test results and prospective data from tests performed during the follow-up period.

Seizures were classified according to the Classification of Seizure Types by the International League Against Epilepsy (ILAE) based on clinical semiology with electroencephalographic support. 22

The study was approved by the Ethical Committee for the Analysis of Research Projects of the Hospital das Clínicas da Universidade de São Paulo (number 3.648.849).

The Fisher exact test was used to compare clinical and complementary data between the groups with and without epilepsy.

All statistical analyses were conducted using Stata SE 15.1 (StataCorp LLC, College Station, TX, USA), and a significance level of 5% was adopted.

## RESULTS

### General findings

A total of 130 patients were assessed, and their age ranged from 1 to 56 years old (mean age 20; 59 (45.4%) were male and 71(54.6%) were female.

### Neurological findings

#### *Epilepsy profile and TAND*

Epilepsy was present in 107 (82.3%) of the 130 TSC patients.

**Figure 1.**
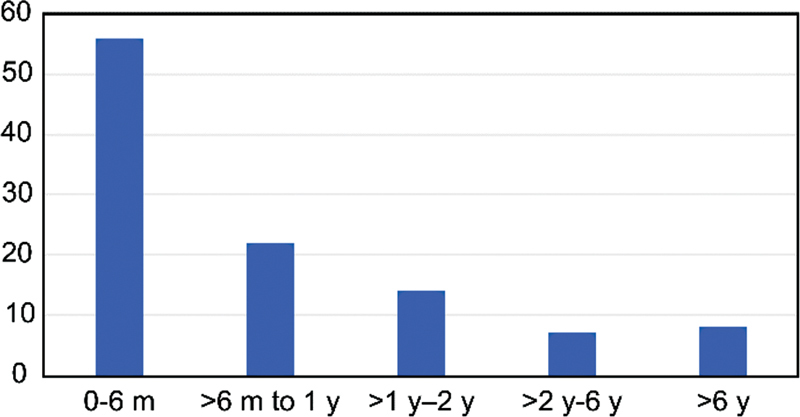
Age of seizure onset.

The distribution of the different patient groups according to the age at the onset of seizures is presented in Figure 1 .

The seizures began before the age of 6 months in 52.3% patients, at between 6 months and 1 year old in 20.5%, and at between 1 and 2 years old in 13%. The proportion of patients with an onset of seizures before 2 years old was 85.9% ( Figure 1 ).

**Figure 2.**
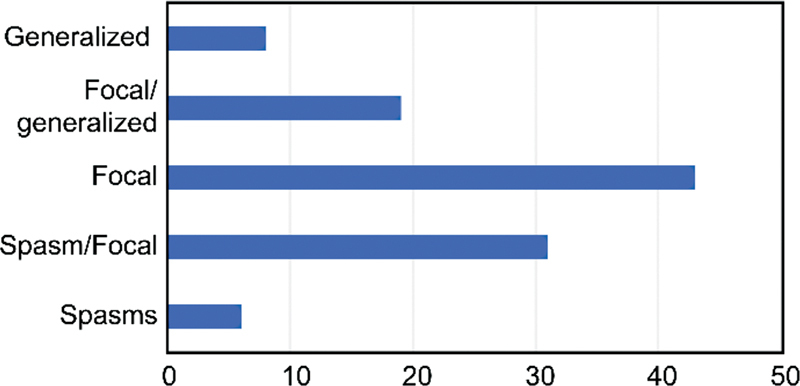
Seizures types.

The predominant type of seizure was focal seizures, reported in 93 (86.9%) patients. The focal seizures occurred in isolation, associated with generalized, or following epileptic spasms. Epileptic spasms were described as the primary type of seizure type in 37 (34.5%) patients ( Figure 2 ). Refractory epilepsy was observed in 59 (50.9%) TSC patients.

The prevalence of TAND was significantly higher, nearly 2-fold increased, in epilepsy patients when compared with the non-epilepsy group ( *p* < 0.001) ( Table 1 ), The symptoms of TAND included autism, intellectual deficits, anxiety disorder, and learning problems.

**Table 1:** Distribution of TSC-associated neuropsychiatric disorders in TSC patients

Finding	Epilepsy		*p* - *value*
	Present	Absent	
TAND	101 (94.4%)	11 (47.8%)	< 0.001*
Normal	6 (5.6%)	12 (52.2%)	

Abbreviation: TAND, TSC-associated neuropsychiatric disorders.

* statistical significance p< 0.05.

#### *Brain magnetic resonance imaging*

The magnetic resonance imaging (MRI) findings related to TSC are described in Table 2 . Five patients were evaluated with brain CT scan, but not with MRI. While most MRI abnormalities (e.g., radial migration lines, subependymal nodules, subependymal giant cell astrocytoma [SEGA]) did not significantly differ in epilepsy versus non-epilepsy patients.

**Table 2:** Neuroimaging findings in TSC patients with and without epilepsy

Finding	Epilepsy		*p-value*
	Present ( *n* = 107)	Absent ( *n* = 23)	
SEN	104 (97.2%)	20 (87.0%)	0.068
CT	103 (96.2%)	18 (78.3%)	0.009*
CERT	18 (16.8%)	0 (0.0%)	0.042*
RML	36 (33.6%)	8 (34.8%)	> 0.999
SEGA	21 (19.6%)	2 (8.7%)	0.365
NP	3 (2.8%)	2 (8.7%)	0.214

Abbreviations: CERT, cerebellar tubers; CT, cortical tubers; NP, not performed; RML, radial migration lines; SEGA, subependymal giant cell astrocytoma; SEN, subependymal nodules.

* statistical significance p< 0.05.

Cortical tubers and cerebellar tubers were significantly more frequent in the epilepsy group, with *p* = 0.009 and 0.042, respectively.

In patients with epilepsy, the neuroimaging studies showed other unusual findings, including previous cerebellar ischemic lesion, cerebellar hypoplasia, hemimegaloencephaly, and hemicortical atrophy.

### Non-neurological manifestations

Typical dermatologic lesions of TSC were present in all patients, with different degrees of appearance and different sizes.

The manifestations of TSC in different systems are described in Table 3 .

**Table 3:** Assessment of TSC patients in different systems

Findings	Epilepsy		*p-value*
	Present	Absent	
**Ophthalmological evaluation**	(n = 85)	(n = 18)	> 0.999
Retinal hamartoma	18 (21.2)	3 (16.7)	
No lesion	67 (78.8)	15 (83.3)	
**Distribution of Pulmonary evaluation**	(n = 66)	(n = 14)	0.002*
MMPH	5 (7.6)	1 (7.1)	
LAM	7 (10.6)	7 (50.0)	
No lesion	54 (81.8)	6 (42.9)	
**Distribution of Cardiological evaluation**	(n = 104)	(n = 22)	0.025*
Rhabdomyoma disappeared	13 (12.5)	4 (18.2)	
Rhabdomyoma present	38 (36.5)	2 (9.1)	
No lesion	53 (51.0)	16 (72.7)	
**Distribution of Renal evaluation**	(n = 106)	(n = 23)	0.838
Renal Cysts	8 (7.6)	3 (13.0)	
AML	58 (54.7)	12 (52.2)	
Renal cysts/AML	10 (9.4)	2 (8.7)	
No lesion	30 (28.3)	6 (29.1)	

Abbreviations: AML, angiomyolipoma; LAM, lymphangioleiomyomatosis; MMPH, multifocal micronodular pneumocyte hyperplasia; NP, not performed.

* statistical significance p< 0.05.

Rhabdomyoma was significantly more frequent in the epilepsy group ( *p* = 0.025), while pulmonary manifestations were significantly less frequent in the epilepsy group ( *p* = 0.002).

The frequency of ophthalmological and nephrological findings did not differ significantly between the groups with and without epilepsy.

Other rare findings included two patients with pancreatic neuroendocrine tumors, one patient with peritoneal mesothelioma, one with adrenal angiomyolipoma, and one with multiplex cutaneous angiomyolipomas.

## DISCUSSION

### Epilepsy and TAND

In the present study of Brazilian TSC patients, epilepsy was present in 82.3% of the cohort, which is similar to previous literature data (83.6%). 23 In our patients, seizures typically began before 2 years old (85.9% of cases), once again in line with other relevant TSC studies. 23 24

The seizures were predominantly focal (86.9%) and occurred in association with other types of seizures ( Figure 2 ). The proportion of predominance of focal seizures is higher than the 67.5% reported in the literature. 23 Epileptic spasms occurred in 37 (34.5%) patients, which is comparable to other reports. 23 24 In another study, among the 76% of TSC patients with epilepsy, 55% had focal seizures, and 57% had epileptic spasms. 25

Refractory epilepsy, as defined by the International League against epilepsy (ILAE), 26 was identified in 59 patients (55.1%). This frequency is somewhat expected as the majority of the patients had an onset of seizures before 2 years old, and it is known that early seizure onset contributes to an increased risk of refractoriness. 27 28

The frequency of refractory epilepsy (for all type of seizures) in the literature for TSC is 62.5% 24_,_ slightly higher than what was observed in our patients. On the other hand, in a large of cohort of 1,852 patients, lower frequency of refractory epilepsy (38%for focal seizures of and 15.5% for epileptic spasms) was attributed to an early diagnosis in a higher number of young patients enrolled, and both diagnosis and management were made in specialized reference centers. 23

Our hospital, as a tertiary hospital, has high complexity cases from throughout Brazil, and some with a late diagnosis of epilepsy, which may impact the number of cases of refractory epilepsy.

Given that TAND symptoms were significantly more prevalent in the epilepsy group (94.4 versus 47.8% in the group without epilepsy), our data reinforce the impression that epilepsy may play a key role for the development of neuropsychiatric manifestations in TSC. In addition, in the group without epilepsy, 52.1% of the patients had no neuropsychiatric manifestations. Moreover, TAND-related symptoms, when present, were sometimes reported as mild and including only learning difficulties and mild anxiety. Thus, interventions to control seizures may improve developmental outcomes and prevent the development of neuropsychiatric symptoms 28 .

The timing of seizure onset, as well as the frequency of seizures and the presence of infantile spasms, are predictive factors for future development. 30 31 In our series, 85.9% had onset of seizures before 2 years old, and 72.8% mainly before 1 year old, some of them without a preventive or early treatment, as they were initially followed-up in other services around Brazil. Preventive treatment strategies prior to the onset of epilepsy have increased in recent years. Treatment is recommended at the earliest sign of epileptogenesis, demonstrated by abnormal eletroencephalography. 30 31 Studies have demonstrated the role of preventive treatment in reducing the frequency of intellectual deficits. 32 33 In the EPISTOP Study, 101 infants were screened, and it was demonstrated that among patients who received preventive treatment, only 33% presented developmental delays at 2 years old, compared with 50% of those who received conventional treatment. This difference suggests that this preventive strategy may change the natural history of epilepsy. 34

### Lesions on brain MRI

In the group with epilepsy, subependymal and cortical tubers were present in 97.2 and 96.2%, respectively, compared with previous rates of ∼ 90%. 35

Cerebellar tubers were significantly more frequent in the epilepsy group ( Table 2 ). Higher numbers of cortical tubers have been related to refractory epilepsy and more severe developmental delay. 36 37 38 39 40

Cortical tubers, with their imbalance of excitation and inhibition, are considered the substrate that may contribute to epileptogenicity. 41 An analysis demonstrated that, in cortical tubers, there is increased expression of glutamate receptors, 42 in different cell types (giant cells and dysplastic neurons), 43 and diminished levels of alpha-aminobutyric acid (GABA) receptors. 42

Cerebellar tubers were present in 18 patients (16.8%). Literature reports indicate that they are present in nearly one-third of TSC patients. 44 As described in previous reports, *TSC2* mutations have been linked to cerebellar tubers as the predominant genotype. 45 46 Cerebellar lesions have been described in TSC patients with more severe neurological involvement (56%), including refractory epilepsy, intellectual disability, and autism disorder, and with a higher cortical tuber count on MRI. 46 In addition, cerebellar atrophy has also been described in 17.1% of TSC patients with cerebellar tubers. 47 One of our patients had had an ischemic cerebellar lesion that was not related to TSC.

The radial migration lines were observed in 33.6% of patients in this series ( Table 2 ). These thin bands of abnormal signal that are radially extended from the juxtaventricular or ventricular white matter lesions are considered histopathologically similar to tubers. 48 49 Radial migration lines may occur in > 90% of TSC patients. 49 They are thin bands of abnormal signal that are radially extended from the juxtaventricular or ventricular white matter, along with cerebral white matter towards the cortical tuber surface or normal cortex. 49 It is possible that a portion of radial migration lines may have been missed as our study included a number of MRI studies performed elsewhere, which may not offer the same resolution and quality as our in our center.

The prevalence of SEGA in the epilepsy group (19.6%) was similar to that found in the literature, which ranges from 5 to 15%. 35 As 45 out of 86 patients without SEGA (53.3%) are < 9 years old, it is possible that some SEGA may still appear in this group given its preponderance in childhood and adolescence, with a median age at diagnosis of 8 years old. 50

Hemimegaloencephaly, present in one patient with epilepsy, is a rare association with TSC as there are 19 cases reported since the first case report in 1961 51_._ The common denominator of hemimegaloencephaly and TSC may be activation of the mTOR pathway. 51

### Non-neurological manifestations

The occurrence of ophthalmological and nephrological findings was not significantly different in the groups with and without epilepsy ( Table 3 ). Retinal abnormalities (hamartomas) were present in 21.2% of the ophthalmological examinations performed in the epilepsy group ( *n =* 85) and 16.7% of the ophthalmological examinations ( *n* = 18) performed in the group without epilepsy ( *n* = 18); in comparison, the rate of retinal lesions described in the literature ranges from 30 to 50%. 13 The fact that > 20% of our entire cohort did not undergo ophthalmologic examinations may contribute to this low rate of retinal hamartomas.

In the group with epilepsy, angiomyolipomas (AMLs) were present in 64.1% of the renal ultrasounds performed ( *n* = 106), and renal cysts were present in only 10 (9.4%) of the renal ultrasounds performed. In addition to these findings, one patient underwent renal transplant, one underwent partial nephrectomy, and one was recently diagnosed with renal cancer.

In the group without epilepsy, 14 (60.8%) patients had AML, 3 had renal cysts, 1 (4%) had renal carcinoma, and 5 (21,7%) underwent nephrectomy. In this series, nearly one-third of the patients were < 11 years old, suggesting the possibility that they may still develop angiomyolipomas over time. According to Ewalt et al., ∼ 80% of patients with TSC will develop AML, which may be identifiable at ∼ 10 years old. 52 Renal cell carcinoma is reported in < 3%, which is quite similar to our finding. 53

The frequency of pulmonary manifestations was significantly lower in the group with epilepsy. This finding may arise from the fact that the patients in the group without epilepsy had no neurological symptoms but pulmonary and nephrological ones as they were referred by the lung and kidney ambulatory clinic for further investigation in the neurology outpatient group.

Another reason may be related to the age range of the epilepsy group, which included 58 patients from 17 to 45 years old and 45 patients from 1 to 16 years old. It can be assumed that the patients may present with pulmonary lesions such as lymphangioleiomyomatosis (LAM), or multifocal micronodular pneumocyte hyperplasia (MMPH) as they age. In the literature, LAM is described in ∼ 30 to 40% of female patients with TSC and in 10 to 12% of male patients. 13

Rhabdomyomas were found at birth in 51 (48.1%) of the examinations performed in the group with epilepsy (n = 106), 38 (36.5%) of which were still present at the time of the present study. This finding is very similar to the rate of ∼ 47 to 67% described in the literature. 13 Rhabdomyomas occurred significantly more frequently in the group with epilepsy than in the group without epilepsy, which might be linked to some underlying common genetic mechanisms yet to be explored. According to the literature, the frequency of rhabdomyoma is estimated at ∼ 60% 54 55 56 in children with TSC, compared with 18% of adult patients. 54 The age at which rapid regression of cardiac rhabdomyomas occurred has been reported as prior to 6 years old. 56 In the group without epilepsy, rhabdomyomas were still present in 2 out of the 6 patients. These two patients who still presented with rhabdomyoma were 42 and 12 years old, which might suggest that these lesions might not disappear spontaneously.

Our results illustrate the multisystemic impairment at different levels in TSC patients regardless the presence or absence of epilepsy. The degree of organ impairment may depend on the age of the patient, as previously discussed.

In conclusion, the present prospective natural history study of patients with and without epilepsy could demonstrates the following:

- Epilepsy, especially before 12 months old, is related to a greater impact of the disease on neurodevelopment.
- Patients with epilepsy had predominant significant higher proportion of neuropsychiatric associated disorders.
- A higher frequency of cortical tubers and cerebellar tubers may impact neurodevelopment.
- Cortical tubers identified on MRI may be considered biomarkers for epilepsy and neurodevelopmental symptoms.
- Cerebellar tubers may play a role for the severity of epilepsy and increased risk of TAND.
- Although the number of patients without epilepsy in the present study was small, it was possible to observe a reduced frequency of TAND symptoms in this group.

Given the possibility of early seizure control improving the neurodevelopment outcomes in TSC, it is necessary to be aware of all potential clinical manifestations in these patients, which eventually may allow clinicians to offer an accurate diagnosis and appropriate treatments.
